# Recombinant thrombomodulin protects against LPS‐induced acute respiratory distress syndrome via preservation of pulmonary endothelial glycocalyx

**DOI:** 10.1111/bph.15153

**Published:** 2020-07-14

**Authors:** Kodai Suzuki, Hideshi Okada, Genzou Takemura, Chihiro Takada, Hiroyuki Tomita, Hirohisa Yano, Isamu Muraki, Ryogen Zaikokuji, Ayumi Kuroda, Hirotsugu Fukuda, Ayane Nishio, Shigeo Takashima, Akio Suzuki, Nagisa Miyazaki, Tetsuya Fukuta, Noriaki Yamada, Takatomo Watanabe, Tomoaki Doi, Takahiro Yoshida, Keisuke Kumada, Hiroaki Ushikoshi, Shozo Yoshida, Shinji Ogura

**Affiliations:** ^1^ Department of Emergency and Disaster Medicine Gifu University Graduate School of Medicine Gifu Japan; ^2^ Department of Internal Medicine Asahi University School of Dentistry Mizuho Japan; ^3^ Department of Tumour Pathology Gifu University Graduate School of Medicine Gifu Japan; ^4^ Division of Genomics Research, Life Science Research Center Gifu University Gifu Japan; ^5^ Department of Pharmacy Gifu University Hospital Gifu Japan; ^6^ Division of Clinical Laboratory Gifu University Hospital Gifu Japan

## Abstract

**Background and Purpose:**

Disruption of the endothelial glycocalyx is causally related to microvascular endothelial dysfunction, a characteristic of sepsis‐induced acute respiratory distress syndrome (ARDS). Recombinant human thrombomodulin (rhTM) attenuates vascular endothelial injuries, but the underlying mechanism remains elusive. Here, we investigated the structural basis and molecular mechanisms of rhTM effects on vascular endothelial injury in a model of sepsis.

**Experimental Approach:**

LPS (20 mg·kg^−1^) was intraperitoneally injected into 10‐week‐old male C57BL6 mice, and saline or rhTM was intraperitoneally injected 3 and 24 h after LPS injection. Using serum and/or lung tissue, histological, ultrastructural, and microarray analyses were performed.

**Key Results:**

Survival rate of rhTM‐treated mice was significantly higher than that of control mice 48 h after LPS injection. Serum concentrations of IL‐6 and high‐mobility group box 1 were lower in the rhTM‐treated group than in the control. Injury to the endothelial glycocalyx in pulmonary capillaries was attenuated by rhTM treatment. Gene set enrichment analysis revealed up‐regulation of gene sets corresponding to cell proliferation/differentiation and anti‐inflammation, such as the TGF‐β pathway, and negative regulation of IL‐6, upon rhTM treatment. Gene expression of heparan sulfate 6‐*O*‐sulfotransferase 1 and endothelial cell‐specific molecule 1 (components of the endothelial glycocalyx) was significantly preserved by rhTM treatment, and their protein expression levels were maintained in endothelial cells.

**Conclusion and Implications:**

Our findings show that rhTM treatment affected inflammation, cell proliferation/differentiation, and glycocalyx synthesis in serum and lung tissue, subsequently attenuating ARDS caused by endothelial injury.

## Graphical abstract

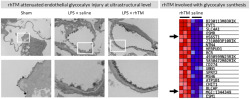

**Abbreviations**
ARDS: acute respiratory distress syndrome
eGC: endothelial glycocalyx
ESM 1: endothelial cell‐specific molecule 1
GO: Gene Ontology
GSEA: gene set enrichment analysis
HMGB1: high‐mobility group box 1
HS6ST1: heparan sulfate 6‐*O*‐sulfotransferase 1
KEGG: Kyoto Encyclopedia of Genes and Genomes
rhTM: recombinant human thrombomodulin

**What is already known**

- The endothelial glycocalyx is injured during endotoxaemia and exacerbates the acute respiratory distress syndrome.

**What this study adds**

- Recombinant human thrombomodulin protects against endothelial injury by accelerating endothelial glycocalyx synthesis.

**What is the clinical significance**

- Recombinant human thrombomodulin could exert beneficial effects against acute respiratory distress syndrome.

## 1 INTRODUCTION

Acute respiratory distress syndrome (ARDS), a clinical phenotype of sepsis, is characterised by a serious inflammatory development comprising neutrophil integration and pro‐inflammatory cytokine production, thereby causing injuries to the alveolar–capillary integrity with high‐permeability and non‐hydrostatic pulmonary oedema (Shao, Tang, Liu, & Zhu, 2016). Under normal conditions, the vascular endothelial glycocalyx covers the surface of endothelial cells and plays a key role in microvascular and endothelial physiology (Chelazzi, Villa, Mancinelli, De Gaudio, & Adembri, 2015). The glycocalyx is an important determinant of vascular permeability (Henry & Duling, 1999; Vink & Duling, 2000). It also regulates neutrophil adhesion and lung injury during sepsis‐induced ARDS (Schmidt et al., 2012), and its disruption by LPS is causally related to microvascular endothelial dysfunction (Inagawa et al., 2018).

Thrombomodulin, which exists on the surface of endothelial cells and contributes to the maintenance of vascular homeostasis (Esmon, 2005), demonstrates an anti‐inflammatory effect by binding to high‐mobility group box 1 (HMGB1), a nuclear architectural chromatin‐binding protein with roles in DNA organisation and transcription regulation (Kadono et al., 2017). Both DNA organisation and transcription regulation play a crucial role in ARDS progression by promoting lung injury (Abeyama et al., 2005; Ito et al., 2008; Ito & Maruyama, 2011). Soluble recombinant human thrombomodulin (rhTM), consisting of all the extracellular domains of thrombomodulin, has been used to treat patients with disseminated intravascular coagulation (DIC) (Kadono et al., 2017). rhTM binds to thrombin to inhibit its procoagulant activity, promotes protein C activation (Kearon et al., 2005; Khorchidi et al., 2002; Tawara, Sakai, & Matsuzaki, 2016), and reduces the secretion of inflammatory cytokines, including IL‐6 and TNF‐α, under septic conditions (Takahashi et al., 2016).

However, to our knowledge, no study has yet investigated whether rhTM attenuates pulmonary endothelial glycocalyx injuries under endotoxaemic conditions. Therefore, this study aimed to evaluate the condition of the pulmonary endothelial glycocalyx after LPS administration in rhTM‐treated mice.

## 2 METHODS

### 2.1 In vivo animal studies

All animal care and experimental procedures in this study conformed to the Guide for the Care and Use of Laboratory Animals (NIH Publication, 8th Edition, 2011) and were approved by the Institutional Animal Research Committee of Gifu University (30‐190, Gifu, Japan). Animal studies are reported in compliance with the ARRIVE guidelines (Kilkenny, Browne, Cuthill, Emerson, & Altman, 2010) and with the recommendations made by the *British Journal of Pharmacology.* All efforts were made to minimise animals' suffering and reduce the number of animals used. Ten‐week‐old male C57BL6 mice (RRID:MGI:5657202; Chubu Kagaku Shizai, Nagoya, Japan) were housed in a colony room maintained at 25°C with a 12:12 h light/dark cycle. For all experiments, animals had free access to powdered mouse chow (CE‐2 Rodent Diet, Nihon CLEA Ltd., 3.40 kcal·g^−1^; 25.1% protein, 4.51% fat, and 49.7% carbohydrate) before and after 16‐h starvation, and tap water, unless otherwise specified. Power analyses (*α* = 0.05, 1 − *β* = 0.95) using an estimated effect size of 1 (based on previous studies, Suzuki et al., 2019) indicated a minimum necessary sample size of five or six animals per treatment group for the experiments described below.

Following 16‐h starvation, mice were injected i.p. with LPS (20 mg·kg^−1^; Millipore Sigma, Burlington, MA) and, in some assays, with recombinant human thrombomodulin (rhTM, 30 mg·kg^−1^; Asahi Kasei Pharma Corporation, Tokyo, Japan) at 3 and 24 h after LPS injection once per day, similar to the clinical use of rhTM in humans. In sham‐operated mice, PBS was injected instead of LPS. Also as a control, PBS was administered instead of rhTM in the same manner. Mice were distributed to each group in a blinded manner. Survival rate was determined every 6 h after LPS administration. Perfusion fixation and blood sampling from the ophthalmic artery were performed under anaesthesia (using a mixture of medetomidine hydrochloride 0.3 mg·kg^−1^, midazolam 4 mg·kg^−1^, and butorphanol tartrate 5 mg·kg^−1^, given i.p.). The anaesthesia was deemed sufficient if the corneal and hind‐paw withdrawal reflexes were absent. Before lung specimens were obtained, the mice were killed by exsanguination from the ophthalmic artery until the righting reflex was lost.

### 2.2 Serum preparation and elisa

Blood samples were collected from six mice, from the buccal artery, which branches off the maxillary artery, allowed to clot at 25°C for 2 h, and centrifuged (2,000 x *g*, 4°C for 20 min). The supernatant (serum) was then collected. Serum IL‐6 was measured using elisa quantitation kits for mouse IL‐6 (Cat No. M6000B, R&D Systems). HMGB1 was measured using elisa quantitation kits for mouse HMGB‐1 by SRL (Hachioji, Tokyo, Japan).

### 2.3 Histopathological examination

Whole right lobes from the lungs of six individual mice 48 h after LPS administration were fixed with PBS containing 10% formalin and embedded in paraffin. Paraffin sections (4 μm) were then deparaffinised and rehydrated. Finally, slides were counterstained with haematoxylin and eosin, and the coverslipped lung sections were scored as follows for pulmonary oedema: 1 = absent; 2 = detectable seroproteinaceous fluid in one to a few alveoli; or 3 = seroproteinaceous fluid‐filled alveoli in a multifocal to coalescing pattern in the lungs. Neutrophilic infiltration was scored: 1 = absent or rare solitary neutrophils; 2 = detectable extravasated neutrophils observed as small loose cellular aggregates in one or a few airways and/or alveoli; 3 = detectable extravasated neutrophils observed as loose to compact cellular aggregates in multiple to coalescing airway and/or alveoli with some effacement of lung architecture; 4 = detectable extravasated neutrophils observed as compact cellular aggregates effacing most adjacent lung architecture (Langlois et al., 2010). These experiments were performed in a blinded manner to avoid bias.

### 2.4 Western blotting

Total protein concentration in tissue lysates was measured using bicinchoninic acid protein assays, and 10 μg of protein was separated by 10% SDS‐PAGE and transferred onto nitrocellulose membranes (Millipore Sigma, Billerica, MA). Membranes were probed with antibodies against syndecan‐1 (ab34164; Abcam, Cambridge, UK) or α‐tubulin (sc‐5546; Santa Cruz Biotechnology, Dallas, TX), and immunoreactive bands were visualised using enhanced chemiluminescence (GE Healthcare UK). Signal intensities were quantified (as arbitrary units) using ImageJ. Western blotting was performed on five independent samples from individual mice from each group.

### 2.5 Scoring of lectin‐staining intensity

For quantitative analysis of glycocalyx injury, scoring of wheat germ agglutinin (WGA, B‐1025‐5; Vector Laboratories, Burlingame, CA, USA) staining intensity was performed using a fluorescence microscope (BZ‐X810, Keyence, Osaka, Japan) and ImageJ software. After deparaffinisation, sections (4 μm) were cut and incubated with WGA biotin conjugate (BK‐1000, Vector Laboratories, Burlingame, CA) at 4°C overnight. After washing with PBS three times, the sections were incubated with DyLight 594 Streptavidin (SA‐5594, Vector Laboratories, Burlingame, CA) at 1:200 dilution in PBS at room temperature for 1 h. After washing with PBS twice, the sections were covered by coverslips. The intensity of WGA expression was scored manually in 10 high‐power fields per sample (*n* = 6 per sample), in the focal plane.

### 2.6 Immunohistochemistry

After deparaffinisation, the sections (4 μm) were cut and incubated with primary antibodies against Ki‐67 (ab16667; Abcam, Cambridge, UK), heparan sulfate 6‐*O*‐sulfotransferase 1 (HS6ST1, bs‐10701R; Bioss Antibodies, Woburn, MA, USA), endothelial cell‐specific molecule 1 (ESM 1; ab103590; Abcam, Cambridge, UK), and the endothelial cell marker CD31 (M0823; Dako, Santa Clara, CA, USA). The target proteins were visualised using the VECTASTAIN Elite ABC system (Vector Laboratories) or the secondary antibodies (Alexa Fluor 488 and 568, Invitrogen) and Hoechst nuclear stain. The immuno‐related procedures used comply with the recommendations made by the *British Journal of Pharmacology.*

### 2.7 Electron microscopy

Electron microscopic analysis of the endothelial glycocalyx was performed as described previously (Inagawa et al., 2018; Okada et al., 2017). Briefly, mice were anaesthetised and then perfused with a solution composed of 2% glutaraldehyde, 2% sucrose, 0.1‐M sodium cacodylate buffer (pH 7.3), and 2% lanthanum nitrate, at a steady flow rate of 1 ml·min^−1^, through a cannula placed in the left ventricle. After mice were killed, lung samples were fixed in a solution without glutaraldehyde and washed thereafter in alkaline (0.03‐M NaOH) 2% sucrose solution. The freeze‐fracture method was used to prepare samples for scanning electron microscopy (S‐4800, Hitachi, Tokyo, Japan), but the method for transmission electron microscopy included embedding the specimens in epoxy resin followed by generation of ultrathin (90‐nm) sections that were stained with uranyl acetate and lead citrate and subjected to transmission electron microscopy analysis (HT‐7700, Hitachi). To prepare samples for conventional electron microscopy, 2.5% glutaraldehyde in 0.1‐M phosphate buffer (pH 7.4) without lanthanum nitrate was used as the fixative.

### 2.8 Microarray analysis

For microarray analysis, lung tissues were obtained from both saline‐ or rhTM‐treated mice 30 h after LPS administration (*n* = 3 each). Total RNA was extracted using a simplyRNA Tissue Kit (Promega, Fitchburg, WI, USA) on a Maxwell RSC instrument. Gene expression analysis of the RNA samples was performed using an Agilent Expression Array (SurePrint G3 Mouse GE 8 × 60K Microarray). The obtained data were analysed and visualised with MeV MultiExperiment Viewer and DAVID. Differentially expressed genes between the rTM‐treated and control saline groups were identified based on the fold change of at least 2 (up‐regulated) or less than 0.5 (down‐regulated) and a *P* value of less than 0.01. Further, gene set enrichment analysis (GSEA) was used to analyse pathway enrichment (http://software.broadinstitute.org/gsea/index.jsp). All microarray data were deposited in the Gene Expression Omnibus (GEO) under Dataset Accession No. GSE130362 (http://www.ncbi.nlm.nih.gov/geo/).

### 2.9 RNA extraction, cDNA synthesis, and quantitative real‐time PCR

RNA was extracted and purified from the lung tissues of six individual mice in each saline‐ and rhTM‐treated group using RNA‐Bee (Tel‐Test, Inc, Friendswood, TX) according to the manufacturer's protocol. RNA concentration and integrity were assessed spectrophotometrically, after which the RNA was reverse transcribed using the high‐capacity cDNA reverse transcription kit (Applied Biosystems, Carlsbad, CA). cDNA was used as a template for quantitative real‐time (qRT)‐PCR, which was performed using TB Green Premix Ex Taq II (Takara Bio) according to the manufacturer's protocol, on a Thermal Cycler Dice TP 990 machine (Takara Bio). The PCR reaction conditions were 50°C for 2 min, 95°C for 10 min, followed by 40 cycles of 95°C for 15 s, plus 60°C for 1 min. The relative quantification of each transcript (*HS6ST1*, *ESM 1*, and *HPSE*) was determined by setting the threshold cycle (Ct) for each sample to reflect the cycle number at which the fluorescence generated within the reaction crossed the threshold level chosen as a point when the amplification was in an exponential phase. *GAPDH* was used as the loading control. The function 2ΔCt was used to determine relative abundance differences, where ΔCt was the difference in Ct values between the compared samples. Primers used in the various PCR reactions are provided in the Table S1.

### 2.10 Data and statistical analysis

Data are presented as means ± SEM. Student's two‐tailed *t* test was used for comparing the two groups, and survival data were analysed using the log‐rank test; *P* < 0.05 was considered significant. All calculations were performed using GraphPad Prism (Ver. 7.02; La Jolla, CA). The data and statistical analysis comply with the recommendations of the *British Journal of Pharmacology* on experimental design and analysis in pharmacology.

### 2.11 Materials

Recombinant human thrombomodulin was provided by Asahi Kasei Pharma Corporation.

### 2.12 Nomenclature of targets and ligands

Key protein targets and ligands in this article are hyperlinked to corresponding entries in http://www.guidetopharmacology.org, the common portal for data from the IUPHAR/BPS Guide to PHARMACOLOGY (Harding et al., 2018), and are permanently archived in the Concise Guide to PHARMACOLOGY 2019/20 (Alexander et al., 2019).

## 3 RESULTS

### 3.1 Treatment with rhTM improved survival after LPS injection

**FIGURE 1:**
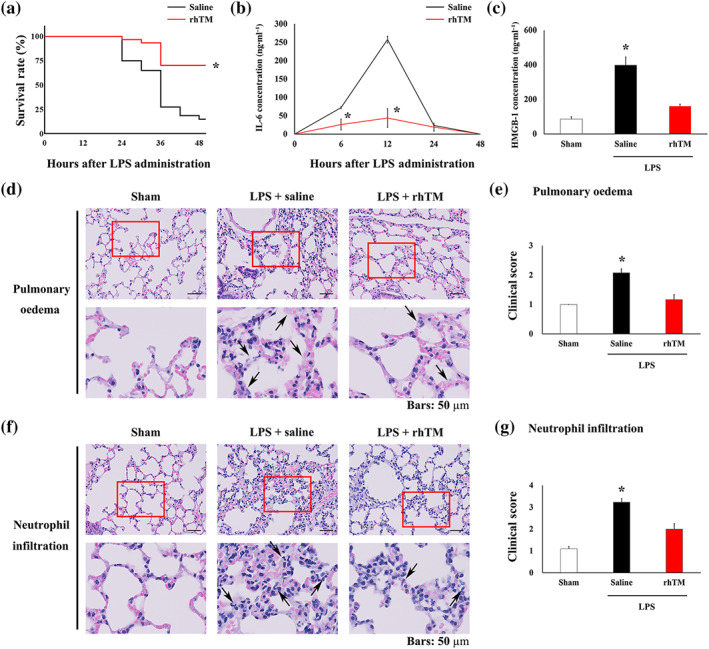
Recombinant human thrombomodulin (rhTM) treatment attenuated LPS‐induced pulmonary injury. (a) Kaplan–Meier survival curves for saline‐injected mice (*n* = 80) and rhTM‐treated mice (*n* = 30) after LPS administration. (b) Serum IL‐6 and (c) high‐mobility group box 1 (HMGB1) concentration was measured in mice using elisa (*n* = 6 each). (d) Haematoxylin and eosin‐stained lung tissues; arrows indicate oedema. (e) Summary data from histological scoring of lung injury caused by pulmonary oedema (*n* = 6 each). (f) Haematoxylin and eosin‐stained lung tissues; arrows indicate neutrophil infiltrations. (g) Summary data from histological scoring of lung injury caused by pulmonary infiltration (*n* = 6 each). In a and b, ^*^ *P* < .05, significantly different from saline‐injected mice. In c, e and g, ^*^ *P* < .05, significantly different from sham or LPS+rhTM

To produce an experimental model of endotoxaemia, LPS (20 mg·kg^−1^) was administered i.p. to 10‐week‐old C57BL6 male mice. The survival rate at 30 h after LPS injection was 65% (52/80) in the control group mice, after which it significantly decreased to 28% (22/80) by 36 h after LPS administration. Alternatively, at 48 h after LPS administration, the survival rate of rhTM‐treated mice was significantly improved over that of control mice (Figure 1a).

In control mice receiving LPS only, IL‐6 levels were significantly raised at 6 h after LPS injection and peaked at 12 h after injection. Thereafter, IL‐6 levels returned to baseline within 48 h after LPS injection (Figure 1b). In rhTM‐treated mice, IL‐6 concentrations were significantly decreased at 6 and 12 h after LPS administration compared with those in control mice. Moreover, serum HMGB1 levels were decreased in rhTM‐treated mice compared with that in untreated mice 48 h after LPS administration (Figure 1c).

To assess pulmonary injury 48 h after LPS injection, we used the previously reported clinical scoring system (Figure 1d–g) (Langlois et al., 2010; Meyerholz et al., 2008). LPS‐injected mice showed increased levels of pulmonary oedema and neutrophil infiltration compared with sham mice. Conversely, rhTM treatment resulted in a significant decrease in both pulmonary oedema and neutrophil infiltration compared with those in saline‐injected mice after LPS administration. These data suggest the attenuation of pulmonary injury by rhTM treatment after LPS administration.

### 3.2 Pulmonary endothelial glycocalyx injury was attenuated in rhTM‐treated mice

**FIGURE 2:**
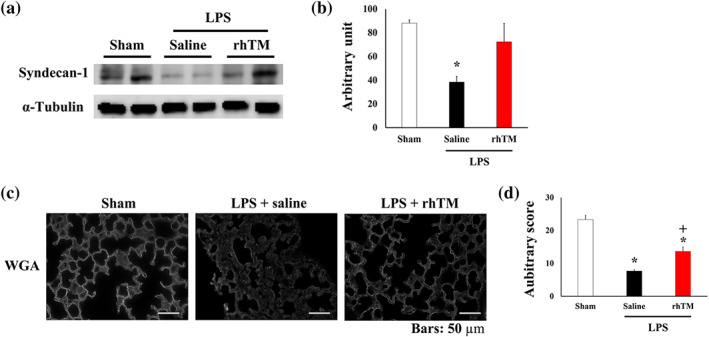
Recombinant human thrombomodulin (rhTM) attenuated LPS‐induced endothelial glycocalyx injury. (a) Representative image of Western blotting of syndecan‐1 in the lungs of sham and saline‐ or rhTM‐treated mice after LPS injection. (b) Densitometry showing relative levels of syndecan‐1 (*n* = 5 per group). ^*^ *P* < .05, significantly different from sham or LPS+rhTM. (c) Lectin, a glycocalyx‐binding glycoprotein, stained by wheat germ agglutinin (WGA). Bars: 50 μm. (d) WGA intensity of saline‐ and rhTM‐injected mouse lungs with and without LPS administration (*n* = 6 each). ^*^ *P* < .05, significantly different from sham mice. ^+^ *P* < .05 significantly different from saline+LPS group

Syndecan‐1, a component of the glycocalyx, was found to be degraded after LPS administration. However, syndecan‐1 degradation was markedly attenuated in rhTM‐treated mice compared with that in saline‐treated mice (Figure 2a,b). To quantitatively assess endothelial glycocalyx injury, we performed intensity measurements using WGA staining, which enabled visualisation of endothelial glycocalyx (Kataoka et al., 2016).

WGA intensity was reduced in saline‐injected mice after LPS administration compared with that in sham mice (Figure 2c,d). After LPS injection, WGA intensity was increased in rhTM‐treated mice compared with that in saline‐treated mice. These results together suggest that endothelial glycocalyx injury in lung capillaries was attenuated by rhTM treatment.

### 3.3 rhTM maintained endothelial glycocalyx structure under endotoxaemic conditions

**FIGURE 3:**
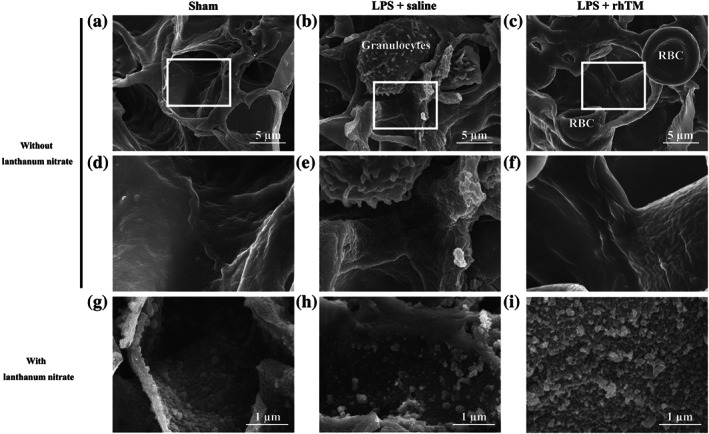
Ultrastructural imaging of pulmonary endothelial injury by scanning electron microscopy. Pulmonary endothelial injury and endothelial glycocalyx injury were attenuated morphologically to a greater extent in recombinant human thrombomodulin (rhTM)‐treated mice than in saline‐injected mice. Conventional imaging (without lanthanum nitrate) of pulmonary endothelium through scanning electron microscopy in (a, d) sham mice and (b, e) saline‐injected and (c, f) rhTM‐treated mice after LPS administration. (d), (e), and (f) are expanded images of the white‐boxed area in (a), (b), and (c) respectively. Glycocalyx imaging (with lanthanum nitrate) through scanning electron microscopy in (g) sham mice and (h) saline‐injected and (i) rhTM‐treated mice after LPS administration. RBC, red blood cell

The ultrastructure of the endothelium and endothelial glycocalyx was analysed using electron microscopy. Conventional scanning electron microscopy results showed the pulmonary capillaries to be of the continuous type, characterised by the presence of an uninterrupted endothelium and a continuous basal lamina, in sham mice (Figure 3a,d). After LPS administration, granulocytes were frequently observed in the pulmonary capillaries, and the inner surface of the vascular endothelium became rough, probably owing to vasculitis by LPS‐induced endotoxaemia (Figure 3b,e). Conversely, rhTM treatment attenuated these effects (Figure 3c,f). To visualise the endothelial glycocalyx, lanthanum nitrate staining was performed. In sham mice, the endothelial glycocalyx was observed to form a continuous structure and cover the inner surface of the vascular endothelium (Figure 3g). After LPS administration, the endothelial glycocalyx was degraded, and its continuous structure was broken in the untreated mice, whereas the continuous structure of endothelial glycocalyx was retained in the rhTM‐treated mice (Figure 3h,i).

**FIGURE 4:**
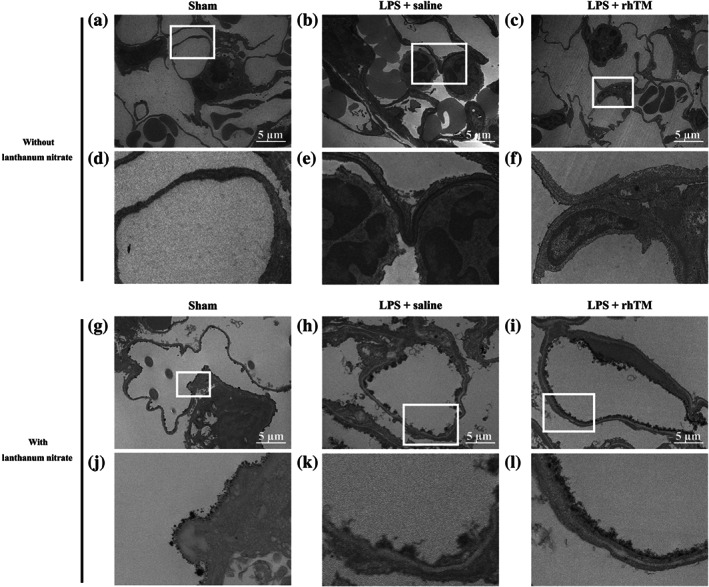
Ultrastructural imaging of pulmonary endothelial injury by transmission electron microscopy. Pulmonary endothelial injury and endothelial glycocalyx injury were morphologically attenuated to a greater extent in recombinant human thrombomodulin (rhTM)‐treated mice than in saline‐injected mice. Conventional imaging (without lanthanum nitrate) of pulmonary endothelium by transmission electron microscopy in (a, d) sham mice and (b, e) saline‐injected and (c, f) rhTM‐treated mice after LPS administration. (d), (e), and (f) are expanded images of the white‐boxed area in (a), (b), and (c) respectively. Glycocalyx imaging (with lanthanum nitrate) by scanning transmission electron microscopy in (g, j) sham mice and (h, k) saline‐injected and (i, l) rhTM‐treated mice after LPS administration. (j), (k), and (l) are expanded images of the white‐boxed area in (g), (h), and (i) respectively

Conventional transmission electron microscopy revealed a smooth inner surface of the vascular endothelium and thin endothelial wall in sham mice (Figure 4a,d). However, after LPS injection, the inner surface of the vascular endothelium became rough, and the endothelial wall became oedematous (Figure 4b,e). Conversely, rhTM treatment attenuated endothelial oedema after LPS administration (Figure 4c,f).

Lanthanum nitrate‐stained transmission electron microscopy revealed that the endothelial glycocalyx indeed forms a continuous structure and covers the inner surface of the vascular endothelium (Figure 4g,j). After LPS injection, the endothelial glycocalyx was peeled away, thereby breaking the continuous structure in untreated mice. Conversely, in the rhTM‐treated group, the continuous structure of the glycocalyx was maintained, and endothelial glycocalyx injury was attenuated (Figure 4h,i,k,l). These results indicate that rhTM treatment attenuated endothelial glycocalyx injury under endotoxaemia.

### 3.4 GSEA following rhTM treatment in the lungs

**FIGURE 5:**
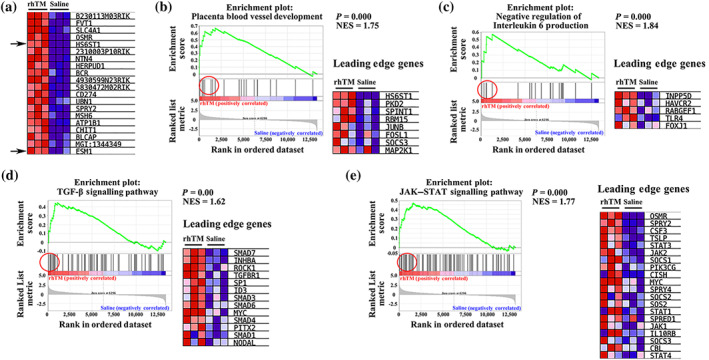
Gene set enrichment analysis. (a) Correlation of heat map and gene list for the top 20 genes of gene set enrichment analysis. (b–e) Enrichment plots and leading‐edge gene lists. (b) Placental blood vessel development, (c) negative regulation of IL‐6 production, (d) TGF‐β signalling pathway, and (e) JAK–STAT signalling pathway. NES, normalised enrichment score

Because rhTM treatment was found to significantly improve survival rate following LPS injection, we next sought to comprehensively determine which genes were affected by rhTM treatment. To this end, GSEA was performed in the control saline‐ and rhTM‐treated groups. The expression of *HS6ST1* and *ESM 1* (components of the endothelial glycocalyx) was significantly increased following rhTM treatment, with their expression shown to be the 4th and 20th highest among all 13,337 genes respectively (Figure 5a). To further confirm the GSEA results, qRT‐PCR was performed for *H6ST1* and *ESM* in the saline‐ and rhTM‐treated groups. Results found that the expression of *H6ST1* and *ESM 1* in rhTM‐treated mice was significantly increased compared with that in the control mice. These results were consistent with those obtained via microarray analysis (Figure S1). Moreover, following Gene Ontology (GO) and Kyoto Encyclopedia of Genes and Genomes (KEGG) analysis of GSEA, gene sets associated with placental blood vessel development (Figure 5b), negative regulation of IL‐6 (Figure 5c), and the TGF‐β and JAK–STAT signalling pathway (Figure 5d,e) were significantly up‐regulated in the rhTM‐treated group compared with those in the control group (*P* < 0.01). Conversely, the expression of genes associated with glycosaminoglycan degradation was not found to differ significantly between the rhTM‐treated and control mice (Figure S2). Additionally, the qRT‐PCR results revealed no significant differences in the expression of *HPSE* (heparinase), an enzyme that degrades polymeric heparan sulfate molecules, between rhTM‐treated and saline‐injected mice (Figure S3). Together, these results suggest that rhTM treatment may functionally affect anti‐inflammatory, cell proliferation or differentiation and glycocalyx synthesis pathways.

### 3.5 Ki67, HS6ST1, and ESM 1 expression in pulmonary capillaries

**FIGURE 6:**
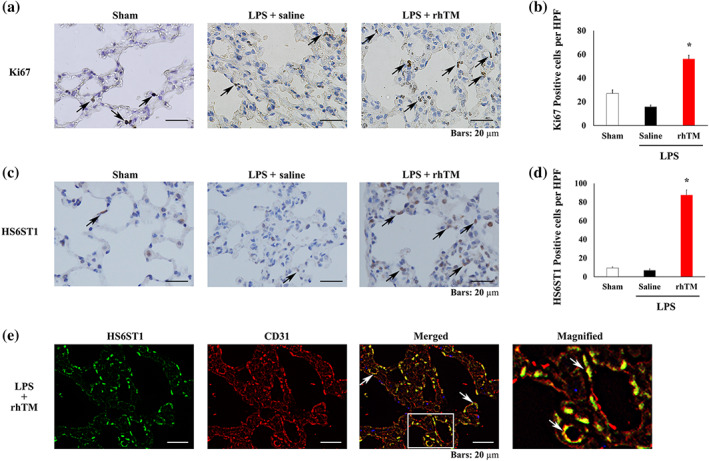
Immunohistochemical analysis of Ki67 and heparan sulfate 6‐*O*‐sulfotransferase 1 (HS6ST1). (a) Immunohistochemical analysis of Ki67 in the lungs of sham mice and saline‐injected and recombinant human thrombomodulin (rhTM)‐treated mice after LPS administration. Arrows indicate Ki67‐positive cells. (b) Graph showing Ki67‐positive cell numbers (*n* = 6 each). ^*^ *P* < .05, significantly different from sham mice. (c) Immunohistochemical analysis of HS6ST1 in the lungs of sham mice and saline‐injected and rhTM‐treated mice after LPS administration. Arrows indicate HS6ST1‐positive endothelial cells. (d) Graph showing HS6ST1‐positive cell numbers (*n* = 6 each). ^*^ *P* < .05, significantly different from sham mice. (e) Double immunofluorescence analysis of HS6ST1 and CD31. The magnified image is the expanded view of the white‐boxed area in the merged image. HPF, high‐power field

**FIGURE 7:**
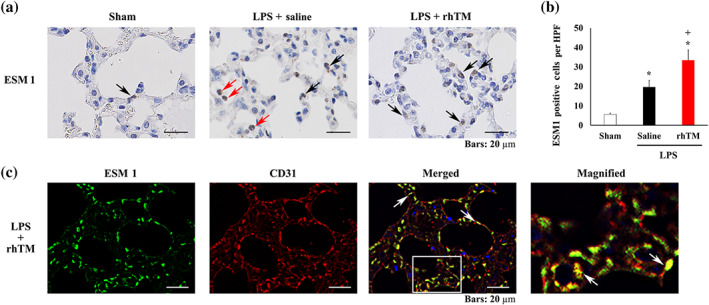
Immunohistochemical analysis of endothelial cell‐specific molecule 1 (ESM 1). (a) Immunohistochemical analysis of ESM 1 in the lungs of sham mice and saline‐injected and recombinant human thrombomodulin (rhTM)‐treated mice after LPS administration. Black arrows indicate ESM 1‐positive endothelial cells. Red arrows indicate ESM 1‐positive inflammatory cells. (b) Graph showing ESM 1‐positive cell numbers (*n* = 6 each). ^*^ *P* < .05, significantly different from sham mice. ^+^ *P* < .05, significantly different from saline+LPS group. (c) Double immunofluorescence analysis of ESM 1 and CD31. The magnified image is the expanded view of the white‐boxed area in the merged image. HPF, high‐power field

Next, to determine the cell proliferation and differentiation capacity, as well as the cellular distribution of HS6ST1 and ESM 1 proteins, immunohistochemical analysis was performed. Specifically, Ki67 expression, a cell proliferation and differentiation marker, was found to be increased in rhTM‐treated mice, compared with the sham and saline‐treated mice (Figure 6a,b). Moreover, HS6ST1 expression was observed in several cell types including inflammatory and endothelial cells. However, its expression in endothelial cells was particularly increased in rhTM‐treated mice compared with sham and saline‐treated mice (Figure 6c,d). To provide further confirmation, double immunostaining for HS6ST1 and CD31, an endothelial cell marker, was performed and HS6ST1 and CD31 were found to be co‐localised in rhTM‐treated mice (Figure 6e). Further, ESM 1 was also expressed in endothelial cells of all experimental groups and in several cell types including inflammatory cells. However, the highest number of ESM 1‐positive endothelial cells was observed in rhTM‐treated mice (Figure 7a,b); meanwhile, both ESM 1 and CD31 were found to be co‐localised in lungs of mice treated with rhTM (Figure 7c). Taken together, these results suggest that rhTM treatment may increase cell proliferation and differentiation and that glycocalyx synthesis may be promoted by HS6ST1 and ESM 1.

## 4 DISCUSSION

This study revealed that rhTM treatment attenuated pulmonary endothelial glycocalyx injuries in mice after LPS administration as compared with that in saline‐treated WT mice. The endothelial glycocalyx covers the surface of healthy vascular endothelial cells and plays an important role in vascular homeostasis (Becker, Chappell, & Jacob, 2010; Chelazzi et al., 2015; Luft, 1966; Okada et al., 2017; Rehm et al., 2004; Reitsma, Slaaf, Vink, van Zandvoort, & Oude Egbrink, 2007; Woodcock & Woodcock, 2012). Previously, it was reported that intact glycocalyx is protective against endothelial disorders (Frati‐Munari, 2013; Gunst et al., 2013). Although this has been applied in specific clinical therapeutic strategies to treat sepsis through endothelial glycocalyx protection (Liu et al., 2018; Wang et al., 2016), these beneficial effects remain controversial and the associated mechanisms had yet to be characterised. Our findings suggest that rhTM may be involved in this glycocalyx protective effect as well as in glycocalyx synthesis.

### 4.1 rhTM accelerates glycocalyx synthesis

Endothelial glycocalyx is synthesised by and covers the surface of endothelial cells in vitro as shown by experiments using HUVEC (Martin, Liberati, & Diebel, 2017). Therefore, the restoration and proliferation of endothelial cells are assumed to be closely related to endothelial glycocalyx synthesis, under septic conditions. The current study revealed accelerated endothelial cell proliferation in rhTM‐treated mice, as supported by GO analysis, which suggested the gene set of TGF‐β signalling pathway and JAK–STAT pathway to be up‐regulated in rhTM‐treated mice. Because the TGF‐β signalling pathway and JAK–STAT pathway contribute to cell proliferation, survival, and differentiation (Constantinescu, Girardot, & Pecquet, 2008; Haan, Kreis, Margue, & Behrmann, 2006; Herpin & Cunningham, 2007; Ikushima & Miyazono, 2010; Murray, 2007; Schmierer & Hill, 2007; Yu, Pardoll, & Jove, 2009), these pathways are expected to be related to survival and restoration of endothelial cells under septic conditions. However, growth factors such as FGF‐10, which occupy roles in cell tissue morphogenesis especially lung development, were recently reported to require heparin sulfate for their function (Chan, Price, & Pratt, 2017; Knelson, Nee, & Blobe, 2014). In addition, FGF signalling has been reported to mediate pulmonary endothelial glycocalyx reconstitution (Yang et al., 2017).

We found that rhTM treatment up‐regulated the gene set associated with placental blood vessel development, including the heparan sulfate 6‐*O*‐sulfotransferase 1 (HS6ST1) gene. This gene is a member of the heparan sulfate biosynthetic enzyme family (Habuchi, Habuchi, & Kimata, 2004) and, as such, is essential for heparan sulfate synthesis, one of the primary components of the endothelial glycocalyx (Reitsma et al., 2007). As HS6ST1 deficiency is lethal in mice primarily during the later embryonic stages, resulting in abnormal angiogenesis in the labyrinthine zone of the placenta along with aberrant lung morphology similar to that observed in pulmonary emphysema (Habuchi et al., 2007), *HS6ST1* is considered to be essential for angiogenesis also in the pulmonary circulation. Furthermore, cell surface heparan sulfate proteoglycans interact with a myriad of growth factors, receptors and extracellular matrix proteins, resulting in regulation of many receptor–ligand interactions (Habuchi et al., 2004). Thus, heparan sulfate has important functions in a variety of developmental, morphogenetic and pathogenic processes (Habuchi et al., 2007). Heparan sulfate biosynthetic enzymes play important roles in producing numerous distinct heparan sulfate fine structures that perform multiple biological activities. Heparan sulfate is also a component of the endothelial glycocalyx and has been recently reported to be associated with endothelial glycocalyx regeneration (Mensah et al., 2017),

Our current study also indicated that ESM 1 expression was increased in rhTM‐treated mice. ESM 1 is a dermatan sulfate proteoglycan that is principally expressed in pulmonary microcirculation as one of the glycocalyx components (Bechard et al., 2000; Lassalle et al., 1996; Orbegozo et al., 2017; Zhang et al., 2012). ESM 1 expression increases in the presence of pro‐angiogenic growth factors, such as FGF‐2 or VEGF (Aitkenhead et al., 2002; Rennel et al., 2007). It also contributes to the pathophysiology of ARDS (Bull, Clark, McFann, Moss, National Institutes of Health/National Heart, & Blood Institute, 2010; Cross & Matthay, 2011; Orbegozo et al., 2017; Tang et al., 2014; Thille et al., 2013). Therefore, not only heparan sulfate and dermatan sulfate proteoglycan but also several glycosaminoglycans such as hyaluronic acid and sialic acid are considered to be endothelial glycocalyx components. Further, the up‐regulation of *HS6ST1* and *ESM 1*, upon rhTM treatment, is suggested to contribute to endothelial glycocalyx biosynthesis. However, rhTM does not accelerate glycosaminoglycan degradation.

### 4.2 rhTM inhibits glycocalyx injuries through its anti‐inflammatory effects

The pulmonary endothelial glycocalyx becomes injured by inflammatory conditions such as sepsis (Inagawa et al., 2018). Therefore, inhibition of inflammation may protect the endothelial glycocalyx structure (Fukuta et al., 2019; Suzuki et al., 2019). Moreover, excessive secretion of IL‐6 causes cellular injury during sepsis (Tanaka, Narazaki, & Kishimoto, 2014). The present study revealed that serum IL‐6 concentration was reduced by rhTM treatment, consistent with the gene set analysis results of negative regulation of IL‐6 production by rhTM.

Although the beneficial effects of rhTM, used in clinical conditions, remains controversial (Vincent et al., 2019; Yamakawa et al., 2016; Yoshihiro et al., 2019), rhTM treatment is known to decrease administration of albumin in patients with severe sepsis‐induced DIC (Hagiwara, Tanaka, Uemura, Matsuda, & Kimura, 2016). Hence, this phenomenon may be involved in decreasing vascular permeability via glycocalyx protection induced by rhTM treatment.

Moreover, the current study showed that rhTM can inhibit serum HMGB1 concentration during endotoxaemia. Because HMGB1 induces the secretion of IL‐1β, TNF‐α, and IL‐6 from several types of inflammatory cells, including neutrophils and macrophages, it plays an important role in initiating and maintaining the amplification of the inflammatory cascade (Fu et al., 2016; Takahashi et al., 2016), consistent with previous reports that suggested that the N‐terminus of thrombomodulin binds to and decomposes HMGB1, subsequently exerting an anti‐inflammatory effect (Abeyama et al., 2005; Ito et al., 2008; Ito & Maruyama, 2011).

### 4.3 Study limitations

Sepsis is an extremely complex condition in humans compared with simple endotoxaemia in an experimental model. Because our focus in this study was to investigate the direct relationship between endothelial glycocalyx injury and septic vasculitis, we used an endotoxaemia model that does not reflect certain typical septic conditions such as bacterial infection. Thus this would represent a limitation of our current study. In this study, rhTM was administered at a high dose of 30 mg·kg^−1^, because the rhTM used was the human, rather than murine, recombinant form. In addition, the route of administration was i.p. and not i.v., as would be typical in a clinical setting.

Although it was shown here that rhTM up‐regulates genes involved in glycocalyx synthesis in the endothelium, which translates to improved glycocalyx coverage and overall survival in LPS‐induced ARDS, further confirmatory studies, including the use of knockout mice, are necessary to fully elucidate the precise mechanisms that allow rhTM to promote endothelial glycocalyx synthesis under conditions of sepsis.

In conclusion, our data has shown that rhTM treatment protected the endothelial glycocalyx from endotoxaemia‐induced lung injury in mice. This mechanism may reduce the damage associated with inflammation and acceleration of the biosynthesis of the glycocalyx itself. As rhTM is currently used in clinical applications, it may be considered as a novel strategy against septic vasculitis, through its ability to protect the endothelial glycocalyx.

## CONFLICT OF INTEREST

None.

## AUTHOR CONTRIBUTIONS

K.S. and H.O. wrote the manuscript. C.T. prepared the samples for transmission electron microscopy imaging. G.T., C.T., and N.M. performed the transmission electron microscopy imaging. K.S., C.T., H.F., and A.N. made the sample for scanning electron microscopy imaging. K.S., H.O., I.M., A.K., and H.F. performed the scanning electron microscopy imaging. K.S., C.T., H.Y., A.K., A.N., T.W., and S.Y. performed the animal studies. A.S. and T.W. performed the elisa experiment. R.Z., T.D., and K.K. performed the immunohistochemistry. H.T., H.Y., T.F., and H.U. assessed the histological score and cell counts. H.O. and S.T. performed the microarray analysis. H.O., S.T., and H.T. analysed the gene set enrichment analysis. G.T. and S.O. supervised the animal studies. H.O. and G.T. revised and edited the manuscript. All authors have read and approved the final manuscript.

## DECLARATION OF TRANSPARENCY AND SCIENTIFIC RIGOUR

This Declaration acknowledges that this paper adheres to the principles for transparent reporting and scientific rigour of preclinical research as stated in the *BJP* guidelines for Design and Analysis, Immunoblotting and Immunochemistry, and Animal Experimentation and as recommended by funding agencies, publishers, and other organisations engaged with supporting research.

## Supporting information


**Figure S1.** Supporting Information

**Figure S2.** Supporting Information

**Figure S3.** Supporting Information

**Table S1:** Primers for relative quantification Real time RT‐PCR Analyses

## Data Availability

The microarray data reported in this paper have been deposited in the Gene Expression Omnibus database under the Accession Number GSE 130362 (http://www.ncbi.nlm.nih.gov/geo/).
