# Supplementary figures and images for: Recombinant thrombomodulin protects against LPS‐induced acute respiratory distress syndrome via preservation of pulmonary endothelial glycocalyx
Source: Br J Pharmacol. 2020 Jul 14;177(17):4021–33. doi: 10.1111/bph.15153 (PMC7429482; doi:10.1111/bph.15153)

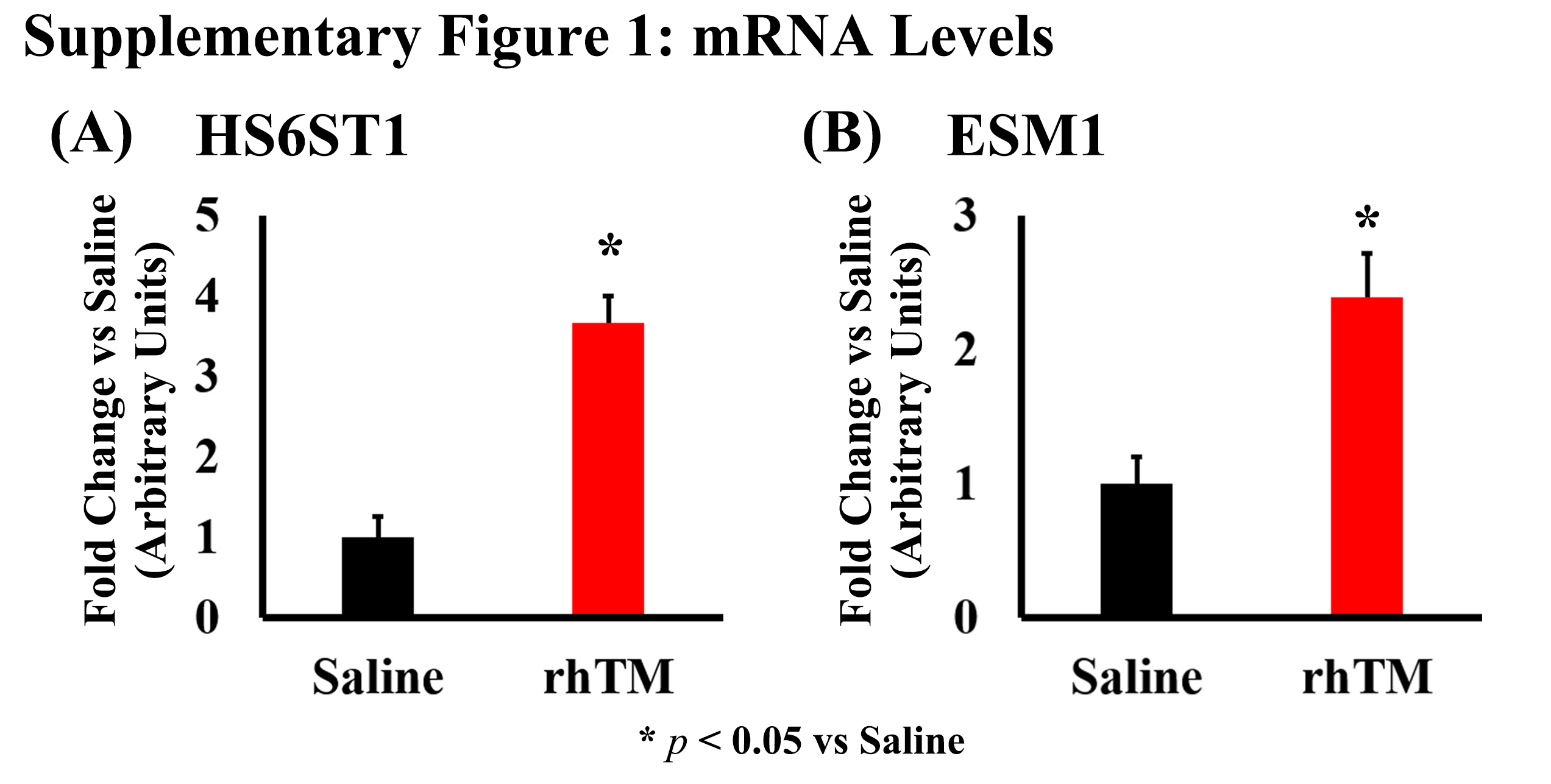

Supplement: Supplementary file 1 — Figure S1. Supporting Information [file BPH-177-4021-s001.tif]

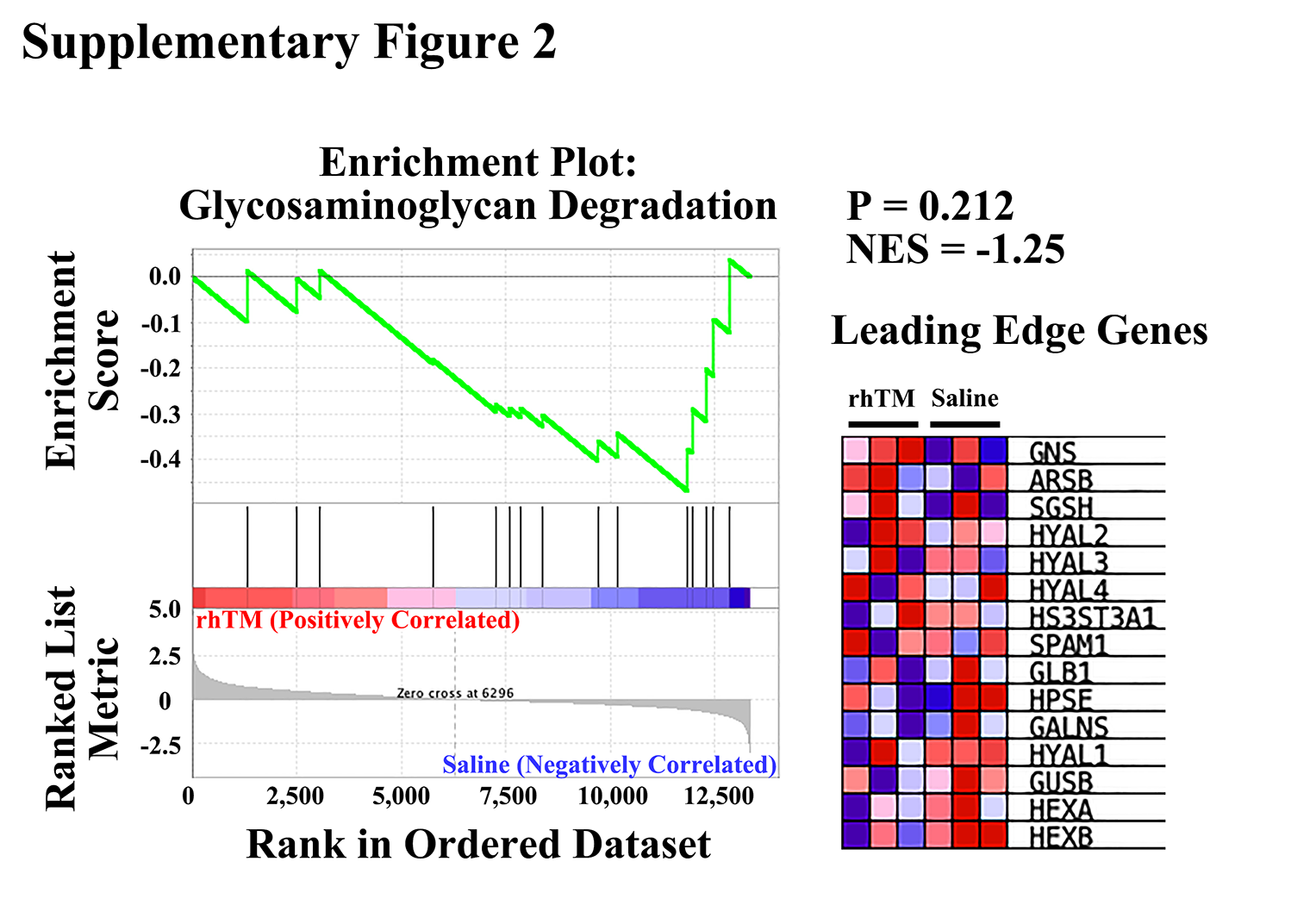

Supplement: Supplementary file 2 — Figure S2. Supporting Information [file BPH-177-4021-s002.tif]

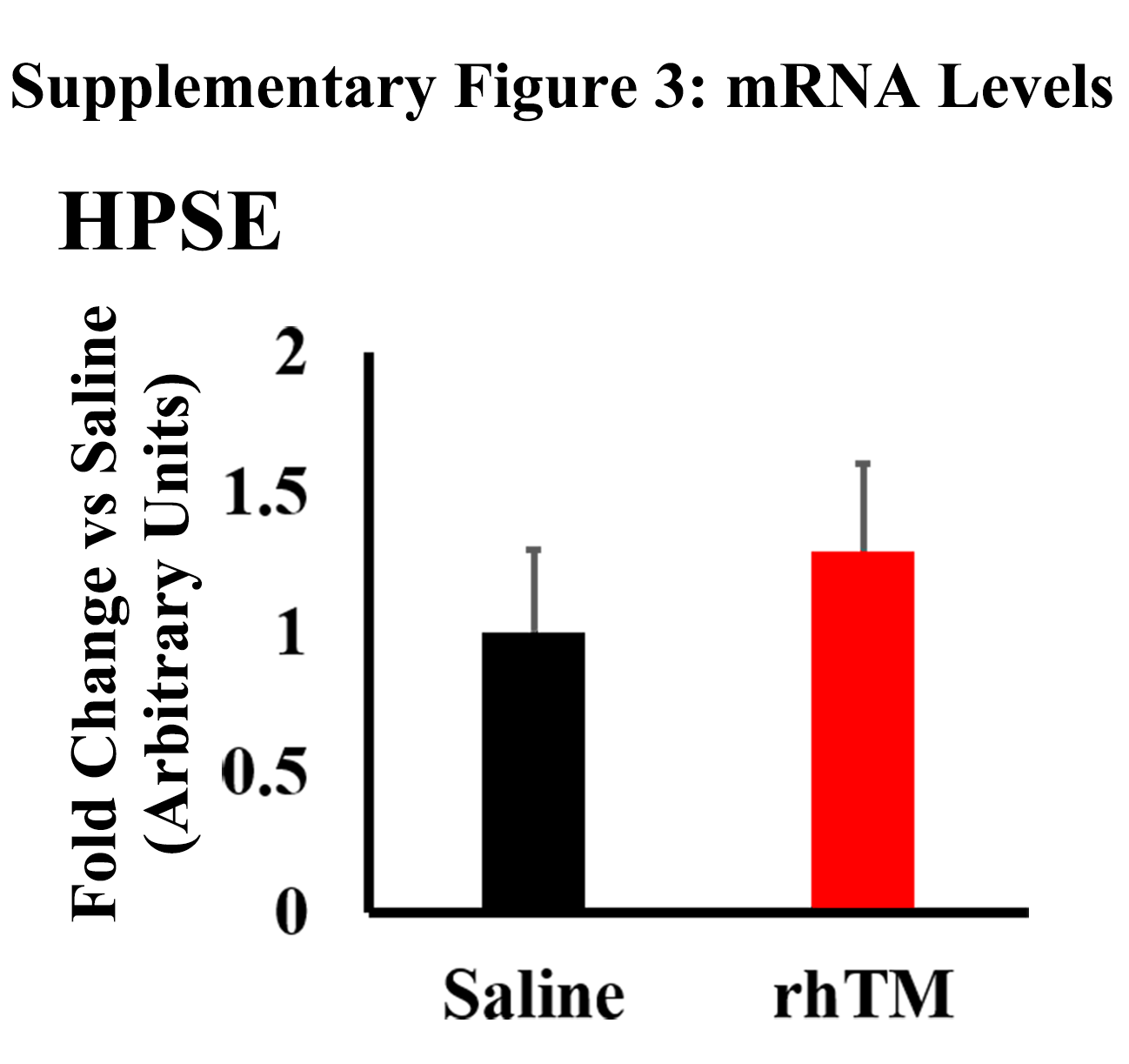

Supplement: Supplementary file 3 — Figure S3. Supporting Information [file BPH-177-4021-s003.tif]
