# Supplementary material for: Recombinant thrombomodulin protects against LPS‐induced acute respiratory distress syndrome via preservation of pulmonary endothelial glycocalyx
Source: Br J Pharmacol. 2020 Jul 14;177(17):4021–33. doi: 10.1111/bph.15153 (PMC7429482; doi:10.1111/bph.15153)
Supplement: Supplementary file 4 — Table S1: Primers for relative quantification Real time RT‐PCR Analyses [file BPH-177-4021-s004.docx]

**Supplementary Table: Primers for relative quantification Real time RT-PCR Analyses**

| ***HS6ST1*** | **F 5’ TGAGAGGAATTTGTTTAGATGCCCAGTTTAG 3’**  **R 5’ TGACAGAAGCAGCAGCAACCAAC 3’** |
| --- | --- |
| ***ESM1*** | **F 5’ CGAGGAGGATGATTTTGGTG 3’**  **R 5’ CTGTCACATATGCCCGACTG 3’** |
| ***HPSE*** | **F 5’ TGAGCTCTGATGTGCTGGAC 3’**  **R 5’ CACATAAAGCCAGCTGCAAA 3’** |
| ***GAPDH*** | **F 5’ ATGTTCCAGTATGACTCCACTCACG 3’**  **R 5’ GAAGACACCAGTAGACTCCACGACA 3’** |
